# The crowding-out effect of sugar-sweetened beverages (SSBs) on household expenditure patterns in Bangladesh

**DOI:** 10.1186/s12889-023-16290-7

**Published:** 2023-07-22

**Authors:** Abul Kalam Azad, Rumana Huque

**Affiliations:** 1 Department of Economics, University of Dhaka, Dhaka-1000, Bangladesh; 2 ARK Foundation, Flat C3 and C4, House # 6; Road # 109, Gulshan 2, Dhaka, 1212 Bangladesh

**Keywords:** Crowding-out, SSB, Logit and multinomial logit model, SUR model, Bangladesh

## Abstract

**Background:**

Consumption of sugar-sweetened beverages (SSBs) or sugary drinks may reduce or even eliminate the household income allocation for other essential commodities. Reducing expenditure for consumption of other household commodities is known as the crowding-out effect of SSB. We aimed to determine the crowding-out effect of SSB expenditure on other household commodities. In addition, we also identified the factors influencing the household's decision to purchase of SSBs.

**Methods:**

We used the logistic regression (logit and multinomial logit models) and the Seemingly Unrelated Regression (SUR) models. In order to find the probability of a given change in the socio-demographic variables, we also estimated the average marginal effects from the logistic regression. In addition, we regressed the SUR model by gender differences. We used Household Income and Expenditure Survey (HIES) 2016 data to estimate our chosen econometric models. HIES is nationally representative data on the household level across the country and is conducted using a multistage random sampling method by covering 46,075 households.

**Results:**

The findings from the logit model describe that the greater proportion of male members, larger household size, household heads with higher education, profession, having a refrigerator, members living outside of the house, and households with higher income positively affect the decision of purchasing SSB. However, the determinants vary with the various types of SSB. The unadjusted crowding out effect shows that expenditure on SSB or sugar-added drinks crowds out the household expenditure on food, clothing, housing, and energy items. On the other hand, the adjusted crowding out effect crowds out the spending on housing, education, transportation, and social and state responsibilities.

**Conclusion:**

Although the household expenditure on beverages and sugar-added drinks is still moderate (around 2% of monthly household expenditure), the increased spending on beverages and sugar-added drinks is a concern due to the displacement of household expenditure for basic commodities such as food, clothing, housing, education, and energy. Therefore, evidence-based policies to regulate the sale and consumption of SSB are required for a healthy nation.

## Background

The consumption pattern of sugar-sweetened beverages (SSBs) or sugary drinks is growing worldwide. Liquids sweetened with various forms of added sugar are commonly known as SSB, such as regular soda, fruit drinks, sports and energy drinks, sweetened waters, pop, cola, tonic fruit punch, lemonade, sweetened powdered drinks, and coffee and tea beverages with added sugars [1]. The SSBs are a main dietary source of added sugar intake, which are rich in calories but poor in nutrients, therefore negatively affecting overall diet quality [2]. A single can of SSB contains around 40 g of free sugars, which is equivalent to about 10 teaspoons of table sugar, and provides calorie equivalent to approximately 200 cal [1]. The increasing trend of SSB consumption is augmenting the risk of noncommunicable diseases (NCDs). The consequences of SSB consumption on health are well documented in the literature [3–8]. Current evidence indicates that frequent SSB intake is linked to gaining weight and could cause obesity and obesity-related diseases such as type 2 diabetes and cardiovascular disease [9, 10]. Hence, the World Health Organization (WHO) recommended to reduce the consumption of added sugars to less than 5% of the daily energy intake (equivalent to around 6 teaspoons of table sugar for adults) of adults and children [11].

Growing consumption of SSBs not only impacts human health but also affects the household resource allocation for basic goods and services. Consumption of SSBs includes both direct (out-of-pocket expenditure) and indirect costs (public health cost) to the consumer and the society in the process of treating obesity, metabolic syndromes (cluster of heart disease, stroke, and type 2 diabetes), and kidney disease [12]. Consumption of SSBs may reduce or even eliminate the household budget allocation for other essential commodities. Reducing expenditure for other household consumption commodities is known as the crowding out effect of SSB. However, the study on the displacement of household income for essential necessities due to SSB consumption is very scanty. Understanding patterns of SSB consumption, any associated socio-demographic characteristics, and the crowding out effect of SSB consumption on necessary commodities are important to design and implement effective public health strategies to lower the consumption of SSBs.

The rate of SSB consumption is alarming in Bangladesh, especially among the youth and children. Evidence suggests that 48% of the school children consumed soft drinks on a daily basis [13], while most of the university students (95.4%) consumed SSBs, and 53.6% reported more than twice a week [14]. We aimed to determine the crowding-out effect of SSB expenditure on other household commodities using secondary data. In addition, we also intended to identify the factors influencing the household's decision to purchase SSB.

## Data, variables, and methodology

### Data and variables

We have wielded Household Income and Expenditure Survey (HIES) 2016 to explore the determinants and crowding-out effect of Sugar-Sweetened Beverages (SSB) or sugary drinks in Bangladesh. HIES data is nationally representative and is collected by the Bangladesh Bureau of Statistics (BBS) at the household level at five-year intervals since 1973–74 [15]. We have used the last round of HIES data, i.e., HIES 2016, which consists of 32,096 rural households and 13,980 urban households across the country. The HIES 2016 portray household consumption patterns for over 140 food items and over 200 non-food items. We have divided the food and non-food items among ten mutually exclusive categories (food, clothing, housing, medicine, education, energy, lifestyle, transportation, social and state responsibility, and durable goods). BBS collects data for food items on a daily and weekly basis, whereas data for non-food items are collected monthly and annually. The detailed survey methodology and design are delineated in the published report of HIES [15].

Sugar-sweetened beverages (SSBs) generally include soft drinks, sugar-added fruit beverages, carbonated drinks, regular sugary soda, and sweetened waters [4]. Tea and coffee are also thought of as beverages if sugar is added during consumption [16], though Zheng et al. (2015) described tea and coffee as substitutes for SSBs [17]. Nevertheless, we defined tea and coffee as SSBs, as most people in Bangladesh add sugar to their tea and coffee consumption.^1^ Since this paper aims to investigate the crowding-out effect of SSB on household expenditure for basic expenditure commodities, we have disaggregated SSB from the food items. Given the nature of the data, we have divided SSB consumption into three categories: soft drinks (Coke, RC, Pepsi, Mojo, sugar-added sherbats, and other soft drinks), other sugar-added drinks (Horlicks, tea, coffee, other sugar-added sherbats), and both. We have considered a household as a beverage and soft drink consumer if any member of the household consumed any beverage drinks. Similarly, if any member of a household purchased any of the other sugar-added drinks or juice is regarded as the consumer of other sugar-added drinks. On the hand, if the household has incurred expenditure on soft drinks and other drinks, it is identified as the consumer of both drinks. The crowding-out effects are investigated for each of these mutually exclusive groups. Besides, since the data are collected daily, monthly, and yearly, we have converted these into monthly for our analysis.

### Method

We have used a logistic regression model to delineate the determinants of consuming sugar-sweetened beverages and other drinks. On the other hand, we have exercised the Seemingly Unrelated Regression (SUR) model to depict the crowding-out effect of sugar-sweetened beverages following a similar kind of study [18]. We first divided the sample households into households that consume any kind of SSBs and households that do not consume any SSBs. We then divided the SSB-consuming households into three mutually independent sub-categories: those that consumed only soft drinks, those that consumed only other sugar-added drinks, and those that consumed both drinks. Therefore, we have used a logistic regression model to estimate the probability of consuming any sugar-added drinks and a multinomial logistic regression model to estimate the probabilities of consuming only soft drinks, only other sugar-added drinks, and only both kinds of drinks controlling a set of socioeconomic characteristics. According to [19], the logistic cumulative distribution function takes the following form.

1 $$P\left(y=1|z\right)=G\left(z^{\prime}\beta \right)=\frac{\mathrm{exp}\left(z^{\prime}\beta \right)}{1+\mathrm{exp}\left(z^{\prime}\beta \right)}$$

where, $G\left(z^{\prime}\beta \right)$ follows the standard logistic distribution function, $y=1$ if any member of a household has positive expenditure on drinks, and $y=0$, if a household does not have any consumption for any drinks. The set of explanatory variables is shown by $z$ vector, including individual and household-level socioeconomic characteristics. The $z$ vector represents the proportion of adult members, the proportion of earners, health condition of the household members, the gender of the household head, religion, access to the refrigerator, residential location, household head migration record, and household income. We have also used categorical variables for household size (number of members from 1–3, 4–6, and 7 and above), education level of the household head (no education, primary education, secondary and higher secondary education, undergraduate, and graduate and postgraduate), and income group (below BDT 5733, 5733–9030, 9031–13,882, 13,883–49,751, and above 49,751).

To address the three groups of drinks consumption, we have estimated the following multinomial logit model to estimate the probabilities of consuming a particular form of drink.

2.1 $$\begin{array}{cc}\left(y=d\vert z\right)=\frac{\exp(z^{\prime}\beta_h)}{1+\sum_{h=0}^{d=2}\exp(z'\beta_h)},&where,h=0,1,2\end{array}$$

where *d* indicates the status of a household consuming a particular kind of drink (i.e., $d=0$ if only soft drinks, $d=1$ if only other sugary drinks, and $d=2$ if only both drinks), since $y$ takes the different values of the categories *d*, the above equation is a multinomial logistic regression model. We considered the households that consume only soft drinks as the base category, and the coefficients of the multinomial logit model are interpreted compared to the reference category. For $d=0$, the model becomes.

2.2 $$\begin{array}{cc}\left(y=0|z\right)=\frac{1}{1+\sum_{h=1}^{d=2}\mathrm{exp}(z^{\prime}{\beta }_{h})},& where\;h=1, 2\end{array}$$

The estimation of the binary model is generally interpreted by calculating the marginal effects from Eq. 2.1 by setting all the predictors at their mean values. But the calculation of marginal effects is complicated and varies from continuous to the discrete independent variable [19]. However, Green (2003) [20] derived the marginal effects more flexibly. The marginal effects for the continuous independent variable:

2.3 $$\frac{\partial P(y=d|z)}{\partial {z}_{ik}}={P}_{ij}({\beta }_{jk}-\sum_{d=0}^{d=2}{\beta }_{dk}{P}_{id})={P}_{ij}\left({\beta }_{jk}-\overline{{\beta }_{i}}\right)$$

On the other hand, the marginal effect can be estimated for a dummy independent variable in the following form [21]:

2.4 $$\frac{\partial P(y=d|z)}{\partial {z}_{ik}}=P\left(y=d|\overline{z }, {z}_{ik}=1\right)-P\left(y=d|\overline{z }, {z}_{ik}=0\right)$$

However, although the marginal effect at the mean value simplifies the estimation, they cannot ensure a unit in the data that indicates the average for all variations. In contrast, Average Marginal Effects (AME) is used more frequently to avoid the above problem [21].

3 $$AME=\frac{1}{n}\sum_{i=1}^{n}\frac{\partial P(y=d|z)}{\partial {z}_{ik}}$$

Thus, AME can be derived by taking the arithmetic mean of each unit of marginal effects.

#### Method for estimating crowding-out

The crowding-out effect of SSB has been defined as the households' reduced consumption of basic necessities due to the consumption of beverages and other sugar-added drinks. On the other hand, crowding out of certain goods and commodities may be derived as the difference of average expenditure shares of different consumption categories between SSBs consumers and non-consumer [18], and/or change in budget share of certain goods and commodities due to one unit change in expenditure on SSBs [18, 22]. Households’ expenditure on SSBs can be zero either because SSB commodities are not in the households’ preference set even if they have enough income; or because households can not afford SSBs due to lack of income. The first situation explains a difference in the consumption pattern of SSBs between SSBs consuming and not consuming households. It also assumes that households that have expenditure on SSBs first make the decision of SSB consumption before making the purchasing decision of other commodities. Therefore, SSB consumption is weakly separable from the consumption of other commodities. It means that household demand for a particular commodity depends on households’ decision of SSB consumption and households’ residual income [18, 23].

The existing literature estimates the crowding out effect in two ways: without controlling (unadjusted) the household socioeconomic characteristics and by controlling (adjusted) them. In the former model, we have compared the mean expenditure share of the households that consume drinks and the households that do not consume any drinks without controlling the socio-demographic variables of the individuals and households. We have also tested the mean differences using the t-test. The negative statistically significant differences are termed as the unadjusted crowding out effect of drink consumption, whereas the positive one increases the expenditure. In contrast, the second model has been estimated based on the household utility maximization problem for the purchasing decision of households between sugar-added drinks and other basic necessities [18]. Therefore, we ran regressions that predicted each expenditure category’s budget share distribution based on the drinking status controlling household socioeconomic characteristics (Eqs. 4.1 and 4.2).

The Quadratic Almost Ideal Demand System (QAIDS) framework [24] is widely used to identify the crowding out effect [22]- estimating the changes in allocated budget shares for different expenditure categories due to the changes in expenditure allocated for SSBs. Due to the absence of direct price information in household level data, one can only estimate conditional Engel curves [23]. Following [22, 23], we also used the conditional Engel curves from the QAIDS framework. The QAIDS permits a specific expenditure category to be either a necessity or luxury by adding a quadratic expenditure variable in the econometric model [22, 24, 25]. The conditional Engel curves can be described for any SSB drinks as follows:

4.1 $${C}_{ij}=\alpha +\gamma {D}_{i}+\theta ln{E}_{i}+\delta {lnE}_{i}^{2}+{z}_{1}\beta +\sum_{d=1}^{8}{\mu }_{d}{Division}_{id}+{e}_{ij}$$

where ${C}_{ij}$ is the expenditure share for expenditure item $j$ for the household$i$. We have divided expenditure items into 10 broad groups (food, clothing, housing, medicine, education, energy, lifestyle, transportation, social responsibility, and durable goods). Expenditure shares are derived after subtracting the expenditure on SSB drinks. ${D}_{i}$ is a dummy variable that takes 1 if a household member purchased any drinks, 0 for otherwise. ${E}_{i}$ denotes the monthly household expenditure after excluding the expenses of drinks, and ${z}_{1}$ are socio-demographic characteristics for the household, including the proportion of adult members, proportion of the earning member, health condition, household size, gender of the household head, religion, education, and household head's major profession. ${Division}_{id}$ controls the spatial fixed effects, that gets 1 if household i is drawn from the residential area *d*, and 0 otherwise. There are 8 administrative division in Bangladesh, and we assume prices are fixed within the division. ${e}_{ij}$ is the stochastic error term.

On the other hand, to estimate the crowding out effect of only soft drinks, only other sugar-added drinks, and only both drinks, we have used the following equation:

4.2 $${C}_{ij}=\alpha +{\sum }_{t=0}^{t=2}{\gamma }_{t}{D}_{it}+\theta ln{E}_{i}+\delta {lnE}_{i}^{2}+{z}_{1}\beta +\sum_{d=1}^{8}{\mu }_{d}{Division}_{id}+{e}_{ij}$$

Equation (4.1) and (4.2) are the systems of conditional Engel curves, and Eq. (4.2) is modified from Eq. (4.1) for the estimation of crowding out for only soft drinks, only other sugar-added drinks and only both drinks. Where $t$ takes all the categories of drinks consumption. ${D}_{it}$ takes 0 if a household purchases only the soft-drink items, 1 if a household purchases only the other sugar-added drinks items, and 2 for only both drinks.

Similar studies of crowding out [18, 22, 23, 26, 27] pointed out the possibility of an endogeneity problem in total expenditure and expenditure of the targeted goods (like soft drinks). Most of the studies of crowding out literature reveal that these variables are endogenous [18, 22, 23]. Therefore, the instrumental variable (IV) method is used to produce consistent and unbiased estimates [22, 23, 25]. Although some candidates like household income, household assets, and adult males to female ratio were used as instrument for total expenditure [18, 22, 23], we have instrumented the household monthly per capita expenditure with monthly per capita income to address the endogeneity problem.

Since the household decision to purchase goods and services is made simultaneously, the expenditure of one item is more likely correlated with other categories. Therefore, we may also assume that the dependent variables will be correlated with the error term of the other equations (contemporaneous correlation) [23]. Each of the equations under the system of Engel curves (Eqs. 4.1 and 4.2) might have SSB drinks consumption as conditioning items with total expenditure and other household characteristics. In addition, as each equation under the system of conditional Engel curves contains the same explanatory variables, the above system of equations (in Eqs. 4.1 and 4.2) is a Seemingly Unrelated Regression (SUR) model A SUR model with IV framework is functionally a Three-Stage Least Squares (3SLS) method [22]. A 3SLS can produce more efficient and consistent estimates compared to other methods under the assumption of homoscedasticity of error term.

## Results

Table 1 portrays household categorization according to household drinking status by rural–urban and income level (for the bottom and top quantiles). It depicts that more than two-thirds (70.12%) of the households consume drinks and juice-related goods. The drinking pattern of SSB is very high among the richest people in the country, indicating that higher-income households have a greater tendency to purchase beverages and sugar-added drinks.

**Table 1:** Distribution of households by drinking status use type and monthly household expenditure

	**All (%)**	**Bottom Quintile (%)**	**Top Quintile (%)**
	(1)	(2)	(3)
**National**			
**Households do not consume any kind of drinks**	29.88	52.49	13.78
Households consume at least one drink	70.12	47.51	86.22
Consume only soft drinks	2.24	1.04	3.23
Consume only other drinks	56.94	44.61	58.58
Consume both	10.95	1.87	24.41
Number of Households	46,075	9215	9215
**Rural**			
**Households do not consume any kind of drinks**	32.86	53.14	15.25
Households consume at least one drink	67.14	46.86	84.75
Consume only soft drinks	2.1	1.06	3.35
Consume only other drinks	56.22	44.08	59.64
Consume both	8.81	1.72	21.77
Number of Households	32,095	7555	5017
**Urban**			
**Households do not consume any kind of drinks**	23.02	49.52	12.03
Households consume at least one drink	76.98	50.48	87.97
Consume only soft drinks	2.58	0.96	3.1
Consume only other drinks	58.57	47.05	57.31
Consume both	15.85	2.53	27.56
Number of Households	13,980	1660	4198

Source: Authors' calculation

It is also evident that urban people are more inclined to consume beverages and other drinks than rural people. In addition, a greater proportion of the households in the top quantile consume SSB compared to the proportion of the households in the bottom quantile, regardless of the households' residential location.

Table 2 describes the summary of major socioeconomic and demographic variables. It shows that households with no experience of SSB consumption earn about Bangladesh Taka (BDT) 4900 and expend BDT 3090 monthly per household member with an average household size of 3.70. On the other hand, households with SSB consumption earn approximately BDT 3860 and spend BDT 4030 monthly per household member, with an average household size of 4.34. Moreover, the households that do not consume drinks have lower monthly per capita expenditure than those who purchase SSB for food and non-food items, including education. Additionally, an average household spends about BDT 271, BDT 168, BDT 243, and BDT 436 monthly for any drinks, only soft drinks, only other sugar-added drinks, and only both drinks, respectively. Table 2 also reports that large households have a greater tendency to spend their income on both food and non-food items.

**Table 2:** Descriptive statistics of household socioeconomic and demographic variables

	**Household Categorization based on drinking status**				
**Variables**	Consuming no SSB	Consuming any SSB	Consuming only soft drinks	Consuming only other sugary drink	Consuming both drinks
Household Monthly Per-capita Income (BDT)	4902.97 (3301.96, 6503.98)	3860.03 (3726.38, 3993.69)	4784.65 (3333.01, 6236.28)	3630.82 (3490.74, 3770.91)	4865.31 (4528.93, 5201.70)
Household Monthly Per-capita Expenditure (BDT)	3088.37 (3033.64, 3143.10)	4028.70 (3991.26, 4066.13)	4544.78 (4311.03, 4778.54)	3738.13 (3699.76 3776.51)	5437.63 (5321.39, 5553.86)
Household monthly food expenditure (BDT)	5122.33 (5059.20, 5185.46)	7784.43 (7730.86, 7838.00)	8465.52 (8076.58, 8854.46)	7261.32 (7208.79, 7313.84)	10,459.66 (10,284.82, 10,634.51)
Household monthly non-food expenditure (BDT)	5620.44 (5437.61, 5803.27)	8529.31 (8402.30, 8656.32)	9921.12 (9112.57, 10,729.67)	7808.67 (7681.58, 7935.74)	12,135.67 (11,700.18, 12,571.17)
Household monthly expenditure on drinks	-	271.21 (268.54, 273.88)	168.76 (154.88, 182.64)	243.52 (240.95, 246.09)	436.16 (427.22, 445.10
Household size	3.70 (3.67, 3.73)	4.34 (4.32, 4.36)	4.38 (4.28,4.48)	4.32 (4.30, 4.34)	4.44 (4.40, 4.49)
Household monthly expenditure on education (BDT)	580.48 (550.61, 610.34)	900.3 (878.03, 922.43)	1201.99 (1043.12, 1360.86)	820.60 (797.85, 843.35)	1272.91 (1200.60, 1345.22)
Male-headed household proportion	0.81 (0.80, 0.81)	0.95 (0.95, 0.96)	0.92 (0.90, 0.94)	0.96 (0.96, 0.96)	0.94 (0.94, 0.95)
Adult member proportion in the household	0.68 (0.67, 0.68)	0.65 (0.65, 0.65)	0.63 (0.62, 0.64)	0.65 (0.65, 0.66)	0.65 (0.64, 0.65)
Adult male member proportion of adults in the household	0.51 (0.51, 0.51)	0.51 (0.51, 0.51)	0.50 (0.49, 0.51)	0.51 (0.51, 0.51)	0.51 (0.51, 0.51)
The proportion of earners in the household	0.32 (0.32, 0.33)	0.32 (0.32, 0.33)	0.31 (0.30, 0.32)	0.32 (0.32, 0.33)	0.33 (0.32, 0.33)
Health status in the household	0.45 (0.44, 0.46)	0.51 (0.50, 0.51)	0.50 (0.47, 0.54)	0.50 (0.50, 0.51)	0.53 (0.51, 0.54)
Migration (either within the country or abroad) status	0.08 (0.08, 0.09)	0.06 (0.06, 0.06)	0.07 (0.05, 0.09)	0.06 (0.05, 0.06)	0.08 (0.07, 0.09)
Having a refrigerator in the household	0.12 (0.11, 0.12)	0.20 (0.19, 0.20)	0.24 (0.21, 0.27)	0.17 (0.17, 0.18)	0.32 (0.31, 0.34)
Education level of the household head	3.50 (3.43, 3.58)	4.58 (4.51, 4.63)	4.94 (4.60, 5.30)	4.40 (4.34, 4.46)	5.47 (5.32, 5.62)

95% confidence interval is in the parenthesis

Similarly, a greater proportion of households consuming SSB are male-headed (95%), compared to the households (81%) with no expenditure on any SSB. However, the proportion of adult members in the households is lower in the households with expenditure on SSB, though the proportion of adult males among adult members and the proportion of earners in the households are approximately identical (Table 2).

Regarding the health consequences, a larger proportion of the households that have spent on SSB (51%) had at least one member who suffered from any chronic disease during the last twelve months than the household with no expenditure on any drinks (45%). Since households that consume SSB may need refrigerators to store them, they may possess refrigerators more likely than households with no drink expenditure. Table 2 depicts that 20% of the households that have purchased SSB possessed at least a refrigerator, whereas only 12% of the households with no expenditure had a refrigerator. Besides, households with SSB consumption allotted a greater amount of their income to education than households without SSB consumption. The heads of households with expenditure on drinks possess a higher level of education (4.58 years) compared to the heads of no-drinking households (3.50 years). In addition, Table 2 also portrays that only heads of both drinking households completed primary education (5.47 years).

We found that the higher proportion of adult members, having sick members, larger household size, urban location of the households, household heads' education, non-agriculture profession of the household head, having a refrigerator, members living outside of the house, and households income positively affect the decision of purchasing SSB (Table 3). If the proportion of adult members is increased by 1%, the average probability of SSB consumption tends to increase by 7%. Similarly, a household with more than 6 members has an 11%, and a household with more than 3 members has a 4% higher probability of purchasing a drink than the reference category (1–3 members). Moreover, if a household is located in an urban area, it has a 7% higher probability of purchasing at least an item of SSB. Besides, having access to a refrigerator increases the 3% probability of consuming SSB for an average household. The higher education and income category are also more likely to influence the household to purchase SSB (Table 3).

**Table 3:** Probability of drinks consumption: average marginal effects

	**Logit Model**	**Multinomial Logit Model**		
(1)	Any kind of drinks (2)	Only Soft Drinks (3)	Only Other Drinks (4)	Only Both Drinks (5)
Proportion of adult members in the household	0.07 (0.03, 0.12)^***^	-0.02 (-0.04, -0.00)^**^	0.02 (-0.02, -0.06)	-0.00 (-0.04, 0.04)
Proportion/Number of earners in the household	0.02 (-0.01, 0.04)	-0.01 (-0.02, 0.01)	-0.03 (-0.07, -0.00)^**^	0.04 (0.01, 0.07)^***^
Health condition	0.04 (0.03, 0.05) ^***^	-0.00 (-0.01, 0.00)	-0.00 (-0.01, 0.01)	0.00 (-0.00, 0.01)
Household size ((1–3) reference category)				
Household size (4–6)	0.04(0.03, 0.06)^***^	0.00 (-0.00, 0.01)	-0.01 (-0.02, -0.00)^**^	0.01 (-0.00, 0.02)^*^
Household size (7 and above)	0.11 (0.08, 0.13)^***^	-0.01 (-0.02, -0.00)^**^	-0.02 (-0.04, 0.00)*	0.03 (0.01, 0.05)^***^
Gender of household head	-0.02 (-0.05, 0.02)	-0.02 (-0.03, -0.01)^***^	0.01 (-0.02, 0.05)	0.01 (-0.02, 0.04)
Religion (muslim = 1, any other category = 0)	-0.08 (-0.09, -0.06)^***^	0.02 (0.01, 0.03)^***^	-0.05 (-0.07, -0.04)^***^	0.03 (0.02, 0.04)^***^
Education of Household head (No education reference category)				
Education of Household head (primary education)	0.04 (0.03, 0.05)^***^	0.00 (-0.00, 0.01)	-0.01 (-0.02, 0.00)^**^	0.01 (-0.00, 0.02)^*^
Education of Household head (secondary education)	0.04 (0.03, 0.05)^***^	0.00 (-0.00, 0.01)	-0.02 (-0.04, -0.01)^***^	0.02 (0.01, 0.03)^***^
Education of Household head (undergraduate)	0.07 (0.07, 0.11)^***^	-0.01 (-0.02, 0.01)	0.01 (-0.02, 0.03)	0.00 (-0.02, 0.02)
Education of Household head (graduate)	0.03 (-0.00, 0.07)	0.00 (-0.01, 0.02)	0.02 (-0.05, 0.01)	0.00 (-0.01, 0.04)
Profession of household head	0.03 (0.02, 0.04)^***^	-0.00 (-0.01, 0.00)	-0.02 (-0.03, -0.01)^***^	0.02 (0.01, 0.03)***
Having refrigerator	0.03 (0.02, 0.05)^***^	0.01 (-0.00, 0.01)^**^	-0.06 (-0.07, -0.05)^***^	0.05 (0.04, 0.07)^***^
Urban (Residential location)	0.07 (0.06, 0.08)^***^	-0.00 (-0.01, 0.00)	-0.04 (-0.05, -0.03)^***^	0.04 (0.03, 0.05)^***^
Migration status in the household	0.03 (0.00, 0.05)^**^	-0.00 (-0.01, 0.01)	-0.01 (-0.03, 0.01)	0.01 (-0.01, 0.03)
Income group ((below BDT 5733) reference category)				
Income group (BDT 5733- BDT 9030)	0.00 (-0.01, 0.05)	-0.00 (-0.01, 0.00)	0.01 (-0.01, 0.03)	-0.00 (-0.02, 0.01)
Income group (BDT 9030 to BDT 13882)	0.05 (0.03, 0.06)^***^	-0.01 (-0.01, 0.00)	-0.02 (-0.03, 0.00)^*^	0.02 (0.01, 0.04)***
Income group (BDT 13882-BDT 49751)	0.05 (0.03, 0.06)^***^	0.00 (-0.00, 0.01)	-0.04 (-0.06, -0.03)^***^	0.04 (0.02, 0.06)^***^
Income group (above BDT 49751)	0.07 (0.05, 0.08)^***^	0.00 (-0.00, 0.01)	-0.07 (-0.08, -0.05)^***^	0.06 (0.05, 0.08)^***^
Wald Chi-Square	1085.46^***^	850.15^***^		
Pseudo R-Square	0.0264	0.0294		

^*^p < 0.10

^**^p < 0.05

^***^p < 0.01; 95% confidence intervals are in parenthesis

Although the proportion of adult members and male-headed households increases the likelihood of the decision to purchase any SSBs or sugary drinks, they decrease the probability for only soft drinks beverages (column 3). However, though the sign and significance for both drinks (column 4) are similar to any drinks (column 1), some deviations are found in only other sugar-added drinks (column 3). For instance, the sign of the refrigerator coefficients is found to be negative. It might happen because the drinks items in other sugar-added drinks may not require the necessity of refrigerators for storage purposes.

Table 3 also reports the Wald chi-squared value associated with the p-value. It presents that the Wald-chi-squared test statistic is statistically significant at 1% level of significance, indicating that the explanatory variables included in the model significantly improve the fitness of the model. Pseudo R-squared values for both logit and multinomial logit model are about 0.03, which may seem to be a lower value, however, logistic regressions generally produce low values of Pseudo R-square.

Table 4 sketches the unadjusted crowding out of the various consumption categories of the households due to the expenditure on SSB. The coefficients of column 2 are considered the reference category for households with having expenditure on beverages and other sugar-added drinks. Since we estimated the results (columns 3, 4, 5, and 6) based on the reference category (households with no SSB consumption), the estimated coefficients are interpreted as percentage point differences. A positive percentage point difference explains that households having expenditure on SSB distributed more share of their expenditure, on average, to that expenditure category, whereas the negative percentage point difference implies that households having expenditure on SSB allocated a smaller share to that particular consumption category. The negative percentage point difference is called the crowding out effect. The detailed unadjusted crowding-out effects are reported in Table 4 from columns 3 to 6.

**Table 4:** Unadjusted Crowding out in the expenditure share due to drinks consumption (Unadjusted difference in the share of consumption expenditure)

Variables	No drinks	Any drinks	Only soft drinks	Only other drinks	Only both drinks
	Mean share (%)	% point difference	% point difference	% point difference	% point difference
**(1)**	(2)	(3)	(4)	(5)	(6)
**Food**	52.66 (52.42, 52.91)	-1.42 (-1.71, -1.13)^***^	-1.93 (-2.90, -0.96)^***^	-1.20 (-1.50, -0.90)^***^	-3.24 (-3.73, -2.76)^***^
**Clothing**	7.22 (7.17, 7.29)	-0.23 (-0.30, -0.16)^***^	-0.41 (-0.66, -0.17)^***^	-0.17 (-0.24, -0.10)^***^	-0.49 (-0.61, -0.37)^***^
**Housing**	9.45 (9.28, 9.63)	-0.74 (-0.94, -0.53)^***^	0.01 (-0.68, 0.71)	-0.90 (-1.11, -0.69)^***^	-0.02 (-0.37, 0.33)
**Medicine**	4.67 (4.55, 4.78)	-0.05 (-0.18, 0.08)	0.43 (-0.03, 0.90)^*^	-0.07 (-0.20, 0.07)	-0.06 (-0.28, 0.16)
**Education**	4.39 (4.27, 4.51)	0.54 (0 .40, 0.69)^***^	1.21 (0.73, 1.70)^***^	0.44(0.29, 0.59)^***^	0.95 (0.70, 1.19)^***^
**Energy**	8.75 (8.67 8.82)	-1.25 (-1.35, -1.16)^***^	-1.46 (-1.80, -1.13)^***^	-1.05 (-1.15, -0.95)^***^	-2.26 (-2.42, -2.10)^***^
**Lifestyle**	4.63 (4.57, 4.68)	0.03 (-0.03, 0.10)	0.08 (-0.15, 0.31)	0.02 (-0.05, 0.09)	0.09 (-0.03, 0.20)
**Transportation**	5.77 (5.66, 5.87)	0.09 (-0.03, 0.21)	0.10 (-0.29, 0.49)	0.01 (-0.11, 0.14)	0.49 (0.29, 0.69)^***^
**Social Responsibility**	1.67 (1.55, 2.78)	0.38 (0.25, .51)^***^	0.50 (0.07, 0.93)^**^	0.27 (0.13, 0.41)^***^	0.94 (0.72, 1.16)^***^
**Durable Goods**	1.42 (1.36, 1.48)	0.31 (0.24, 0.38)^***^	0.58 (0.34, 0.81)^***^	0.21 (0.14, 0.28)^***^	0.78 (0.66, 0.89)^***^

^*^p < 0.10

^**^p < 0.05

^***^p < 0.01; 95% confidence intervals are in parenthesis

According to column 3 of Table 4, expenditure on SSB crowds out household expenditure on food, clothing, housing, and energy commodities of the households. On the other hand, expenditure on SSB is positively associated with expenditure on education, social responsibility, and durable goods. The second column shows the mean share of households without SSB consumption. The third column shows that an average household has shrunk its budget on food by 1.42 percentage points due to the expenditure on any SSB. In addition, the spending on SSB reduces the expenditure on clothing, housing, and energy commodities by 0.23, 0.74, and 1.25 percentage points, respectively, on average. However, the expenditure on an SSB increases the budget allocation on education, social responsibility, and durable goods, on average, by 0.54, 0.38, and 0.31 percentage points, respectively.

Some differences are found in the magnitudes, signs, and significance levels of the coefficients for the SSB expenditure by type. For instance, the coefficients of the housing category for the household with expenditure only soft drinks and both drinks are found positive and negative, respectively, without being statistically significant. The magnitude of food items for the household with having expenditure only on both types of drinks is almost double (-3.24) than the other categories. It implies that expenditure on both kinds of drinks crowds out the larger portion of the food budget for the households than other categories.

Since the estimated results of Table 4 do not control the covariates of the model, the results reported in Table 4, hence, are not the causal impact of the expenditure on SSB on the household's different consumption categories. Table 5 shows the causal effect from the estimation of the SUR model (adjusted crowding-out effect). The results deviate for some types from the unadjusted crowding out due to the inclusion of covariates. Column 2 of Table 5 shows that the budget allocation for any SSB crowds out the expenditure on housing, education, transportation, and social responsibility by 2.30, 0.36, 1.10, and 0.91 percentage points, respectively. However, expenditure on the food item is found to be positively associated with spending on drinks by an amount of 3.04% points.

**Table 5:** Adjusted Crowding out in the expenditure share due to drinks consumption (Seemingly Unrelated Regression (SUR) Model)

	**Household Categorization based on drinking status**			
**Variables**	Any SSB	Only soft drinks	Only other sugary drinks	Both drinks
	% point difference	% point difference	% point difference	% point difference
(1)	(2)	(3)	(4)	(5)
**Food**	3.04 (1.90, 4.19)^***^	5.62 (-7.36, 18.59)	2.484 (0.94, 4.03)^***^	8.63 (5.85, 11.40)^***^
**Clothing**	-0.331 (-1.30, 0.55)	-0.62 (-2.11, 0.87)	-0.16 (-1.12, 0.79)	-0.84 (-2.11, 0.43)
**Housing**	-2.295 (-3.10, -1.49)^***^	-3.70 (-8.74, 1.33)	-2.12 (-3.26, -0.98)^***^	-4.75 (-6.30, -3.19)^***^
**Medicine**	0.06 (-0.17, 0.28)	0.94 (-2.87, 4.75)	0.07 (-0.17, 0.30)	0.08 (-0.77, 0.94)
**Education**	-0.36 (-0.60, -0.20)^***^	0.07 (-2.16, 2.29)	-0.37 (-0.66, -0.09)^**^	-0.53 (-1.27, 0.21)
**Energy**	0.04 (-1.10, 1.17)	-0.25 (-1.04, 0.55)	-0.23 (-0.43, -0.03)^**^	-0.51 (-2.06, 1.04)
**Lifestyle**	-0.02 (-0.83, 0.79)	-0.04 (-0.69, 0.61)	0.06 (-0.85, 0.96)	-0.25 (-1.43, 0.94)
**Transportation**	-1.10 (-1.31, -0.88)^***^	-1.66 (-3.07, -0.25)^**^	-0.98 (-1.18, -0.77)^***^	-2.147 (-2.84, -1.45)^***^
**Social Responsibility**	-0.91 (-1.17, -0.64)^***^	-1.55 (-3.07, -0.03)^**^	-0.75 (-1.07, -0.44)^***^	-1.94 (-2.53, -1.36)^***^
**Durable Goods**	-0.20 (-1.09, 0.68)	0.33 (-1.11, 1.77)	-0.05 (-1.00, 0.90)	-0.15 (-1.46, 1.15)

^*^p < 0.10

^**^p < 0.05

^***^p < 0.01; 95% confidence intervals are in parenthesis

The estimated adjusted crowding out results (column 2) due to the expenditure for SSB by types in household different consumption categories are almost similar to the crowding out effect due to the expenditure on any SSB consumption. However, there are some variations in magnitude, signs, and level of significance among the different types of models, like the unadjusted crowding-out effect. The most apparent difference is found in the magnitude of the food items (8.63) of the household's expenditure on only beverages and other sugar-added drinks.

Table 6 presents the adjusted crowding-out effect on various households' consumption items disaggregated by gender. It shows that male-headed households are more prone to allocate their income to SSB, and therefore crowding out for various food and non-food items is more prevalent among male-headed households than female-headed households. Household income allocation on SSB (column 2) crowds out the spending on housing, education, energy, transportation, and social responsibility on average for male-headed households. However, most of the estimated coefficients for the female-headed households' samples are insignificant, indicating that the spending on SSB does not influence the budget allocation for other items for female-headed households.

**Table 6:** Adjusted Crowding out in the expenditure share due to drinks consumption (Seemingly Unrelated Regression (SUR) Model) with household head gender identity

	**Male**				**Female**			
**Variables**	Any SSB	Only soft drinks	Only other drinks	Both drinks	Any SSB	Only soft drinks	Only other drinks	Both drinks
	% point difference	% point difference	% point difference	% point difference	% point difference	% point difference	% point difference	% point difference
**(1)**	(2)	(3)	(4)	(5)	(6)	(7)	(8)	(9)
**Food**	3.02 (1.64, 4.41)^***^	7.40 (5.49, 9.30)^***^	2.48 (0.48, 4.48)^**^	8.54 (5.10, 11.97)^***^	3.14 (-3.17, 9.45)	10.48 (2.22, 18.73)^**^	1.25 (-6.38, 8.87)	11.13 (2.00, 20.25)^**^
**Clothing**	-0.21 (-1.16, 0.74)	-0.793 (-1.31, -0.28)^***^	-0.28 (-0.48, -0.08)^***^	-0.70 (-2.13, 0.73)	0.63 (-1.46, 2.71)	-1.65 (-4.24, 0.94)	1.11 (-1.97, 4.20)	-0.87 (-3.15, 1.41)
**Housing**	-2.29 (-3.25, -1.33)^***^	-4.55 (-5.92, -3.19)^***^	-2.12 (-3.58, -0.66)^***^	-4.75 (-6.58, -2.93)^***^	-3.65 (-8.79, 1.49)	-3.73 (-10.95, 3.49)	-3.26 (-9.98, 3.47)	-7.06 (-14.14, 0.02)^*^
**Medicine**	0.05 (-0.18, 0.28)	0.40 (-0.45, 1.24)	0.06 (-0.20, 0.32)	0.11 (-0.87, 1.09)	-0.27 (-2.98, 2.44)	-1.83 (-4.89, 1.24)	-0.59 (-4.29, 3.10)	-0.51 (-3.71, 2.68)
**Education**	-0.334 (-0.59, -0.08)^**^	0.47 (-0.44, 1.37)	-0.36 (-0.71, -0.02)^**^	-0.50 (-1.31, 0.32)	-2.39 (-6.65, 1.88)	-1.57 (-6.39, 3.24)	-2.34 (-7.97, 3.29)	-2.57 (-7.51, 2.36)
**Energy**	-0.30 (-0.51, -0.09)^***^	-0.24 (-0.86, 0.39)	-0.22 (-0.46, 0.03)^*^	-0.84 (-1.27, -0.41)^***^	-0.81 (-3.29, 1.67)	-0.32 (-4.92, 4.28)	-0.61 (-3.93, 2.71)	-0.48 (-3.99, 3.03)
**Lifestyle**	0.05 (-0.85, 0.96)	-0.14 (-0.59, 0.32)	0.09 (-0.95, 1.12)	-0.17 (-1.53, 1.19)	1.51 (-0.10, 3.13)*	0.32 (-1.04, 1.67)	1.70 (-0.58, 3.98)	1.38 (-0.22, 2.99)^*^
**Transportation**	-1.09 (-1.31, -0.87)^***^	-1.83 (-2.71, -0.95)^***^	-0.97 (-1.18, -0.75)^***^	-2.10 (-2.83, -1.38)^***^	-0.65 (-3.41, 2.12)	-1.93 (-5.66, 1.81)	0.29 (-3.83, 4.48)	-4.19 (-8.04, -0.33)^**^
**Social Responsibility**	-0.92 (-1.22, -0.62)^***^	-1.85 (-2.63, -1.07)^***^	-0.77 (-1.16, -0.38)^***^	-1.95 (-2.56, -1.33)^***^	-0.10 (-2.74, 2.54)	0.06 (-1.70, 1.83)	-0.31 (-3.80,) 3.19)	-1.27 (-3.63, 1.09)
**Durable Goods**	-0.08 (-1.05, 0.90)	0.13 (-0.31, 0.56)	0.01 (-1.05, 1.07)	-0.03 (-1.54, 1.48)	0.28 (-1.03, 1.60)	-0.55 (-1.85, 0.75)	0.42 (-1.13, 1.97)	1.07 (-0.80, 2.94)

^*^p < 0.10

^**^p < 0.05

^***^p < 0.01; 95% confidence intervals are in parenthesis

In both Tables 5 and 6 (Male sub-sample), the coefficients of food, housing, transportation, and social or state responsibility are highly statistically significant. On the other hand, though the education and energy variables have some significant coefficients, they are not consistent across the different specifications. It implies that households certainly reduce the budget shares of housing, transport, and social and state responsibility, but they remain cautious about education and energy expenditure. In addition, the coefficients in the female sub-sample (Table 6) are mostly insignificant, with few marginally significant coefficients. It indicates that male-headed households displace more of their budget share than female-headed households.

Households may have zero consumption of SSBs due to the lack of income. It implies that poor households may have corner solutions for SSBs consumption. Existing literature also shows that poorer households have significantly lower SSBs consumption than richer households [28, 29]. Therefore, one can assume that consumption for other consumption categories will not be affected by the poorer people. Based on this hypothesis, we estimated the crowding-out effects by dividing the household according to the income quantiles for each of the four SSB categories: any SSBs, only soft drinks, only other sugar-added drinks other than soft drinks, and only both drinks. Results of the crowding out for each of the five income quantiles are reported in Table 7. Obtained results confirmed that the consumption of almost all commodities is unaffected for the household belonging to the 1st income quantile-the poorest households (Table 7). On the other hand, as income rises (quantile 2, 3, 4, and 5), consumption of SSBs displaces the budget shares for the other basic goods and commodities (Table 7).

**Table 7:** Adjusted crowding out in the expenditure shares due to drinks consumption (Seemingly Unrelated Regression (SUR) Model) based on income categories

	**Any Sugar-Added Drinks**					**Only Soft Drinks**				
**Variables**	IQ^i^-1	IQ-2	IQ-3	IQ-4	IQ-5	IQ-1	IQ-2	IQ-3	IQ-4	IQ-5
	% point difference	% point difference	% point difference	% point difference	% point difference	% point difference	% point difference	% point difference	% point difference	% point difference
**(1)**	(2)	(3)	(4)	(5)	(6)	(7)	(8)	(9)	(10)	(11)
**Food**	0.01 (-3.93, 3.943)	1.16 (-0.78, 3.10)	1.56 (0.39, 2.72)***	2.55 (1.53, 3.57)***	5.00 (3.65, 6.34)**	6.20 (-11.38, 23.79)	3.69** (0.15, 7.24)**	2.93 (-0.77, 6.63)	1.89 (-0.84, 4.61)	4.83 (1.37, 8.28)***
**Clothing**	-0.71 (-6.82, 5.40)	-0.00 (-0.57, 0.57)	-0.42 (-0.74, -0.10)**	-0.11 (-0.39, 0.18)	-0.10 (-0.49, 0.29)	-0.63 (-4.35, 3.10)	0.02 (-1.10, 1.15)	0.23 (-0.91, 1.37)	-0.23 (-0.99, 0.54)	-0.06 (-1.10, 0.99)
**Housing**	0.052 (-2.15, 2.26)	-2.02 (-3.35, -0.69)***	-1.89 (-2.68, -1.11)***	-2.33 (-3.15, -1.52)***	-2.42 (-3.63, -1.21)***	-0.72 (-8.31, 6.88)	-4.34 (-6.68, -1.99)***	-3.41 (-5.89, -0.93)***	-2.05 (-4.74, 0.63)	-4.61 (-8.71, -0.51)**
**Medicine**	1.56 (-3.69, 6.80)	-0.99 (-2.00, 0.03)*	-0.42 (-0.91, 0.08)*	0.03 (-0.45, 0.51)	-0.58 (-1.23, 0.08)*	2.09 (-12.51, 16.70)	-1.15 (-2.93, 0.62)	-0.02 (-1.64, 1.59)	0.59 (-0.65) 1.82)	-0.93 (-2.60, 0.75)
**Education**	-1.54 (-4.19, 1.12)	0.44 (-0.62, 1.50)	0.64 (0.04, 1.24)**	0.04 (-0.56, 0.65)	-1.13 (-2.02, -0.24)**	-3.01 (-7.76, 1.73)	2.04 (0.12, 3.96)**	2.55 (0.72, 4.38)***	1.51 (-0.29, 3.31)	0.90 (-1.16, 2.96)
**Energy**	0.96 (-6.81, 8.73)	-0.95 (-1.70, -0.20)**	-1.05 (-1.45, -0.65)***	-1.04, (-1.42, -0.65)***	-0.33 (-0.80, 0.14)	0.98 (-2.41, 4.37)	-0.90 (-2.30, 0.51)	-2.75 (-4.23, -1.26)***	-1.15 (-2.14, -0.17)**	-0.14 (-1.80, 1.51)
**Lifestyle**	0.18 (-1.47, 1.82)	0.53 (0.05, 1.01)**	-0.06 (-0.31, 0.19)	-0.13 (-0.41, 0.16)	-0.13 (-0.56, 0.30)	0.71 (-2.58, 4.00)	0.86 (-0.10, 1.83)*	0.10 (-0.68, 0.88)	-0.35 (-1.11, 0.41)	0.04 (-1.10, 1.18)
**Transportation**	-2.04 (-6.20, 2.12)	0.01 (-0.83, 0.85)	-0.34 (-0.86, 0.19)	-0.94 (-1.49, -0.40)***	-0.90 (-1.75, -0.04)**	-5.41 (-12.50, 1.69)	0.56 (-0.88, 1.99)	-0.07 (-1.66, 1.52)	-1.19 (-2.62, 0.25)	0.612 (-2.13, 3.35)
**Social Responsibility**	-0.82 (-2.60, 0.97)	0.11 (-0.69, 0.90)	-0.01 (-0.46, 0.43)	0.10 (-0.42, 0.63)	-0.73 (-1.52, 0.06)*	-0.79 (-5.15, 3.58)	-1.40 (-2.63, -0.17)**	-0.46 (-1.82, 0.90)	-0.144 (-1.34, 1.06)	-2.60 (-4.45, -0.74)***
**Durable Goods**	-0.13 (-2.79, 2.52)	-0.28 (-0.78, 0.23)	-0.10 (-0.37, 0.17)	-0.11 (-0.38, 0.16)	-0.41* (-0.85, 0.02)*	-0.36 (-3.02, 2.30)	-0.38 (-1.29, 0.53)	-0.04 (-1.19, 1.11)	0.30 (-0.42, 1.02)	1.09 (-0.59, 2.78)
	**Only Other Sugar-Added Drinks other than Soft Drinks**					**Only Both Drinks**				
**Variables**	IQ-1	IQ-2	IQ-3	IQ-4	IQ-5	IQ-1	IQ-2	IQ-3	IQ-4	IQ-5
	% point difference	% point difference	% point difference	% point difference	% point difference	% point difference	% point difference	% point difference	% point difference	% point difference
**(12)**	(13)	(14)	(15)	(16)	(17)	(18)	(19)	(20)	(21)	(22)
**Food**	-0.19 (-2.37, 1.99)	0.48 (-1.45, 2.42)	1.68, (0.54, 2.82)***	2.28 (1.31, 3.24)***	4.43 (3.04, 5.82)***	4.72 (-7.26, 16.70)	1.64 (-1.92, 5.20)	3.52 (0.64, 6.40)**	5.85 (3.59, 8.17)***	9.12 (4.974, 13.27)***
**Clothing**	-0.59 (-1.21, 0.03)*	-0.01 (-0.59, 0.56)	-0.55 (-0.87, -0.22)***	-0.09 (-0.36, 0.18)	-0.01 (-0.41, 0.40)	-1.37 (-5.98, 3.24)	0.54 (-0.57, 1.66)	0.15 (-0.63, 0.92)	-0.47 (-1.11, 0.18)	-0.43 (-1.56, 0.70)
**Housing**	-0.19 (-1.70, 1.33)	-1.41 (-2.73, -0.09)**	-1.76 (-2.55, -0.98)***	-2.03 (-2.82, -1.24)***	-1.88 (-3.10, -0.67)***	0.10 (-7.66, 7.87)	-3.36 (-6.03, -0.68)**	-4.80 (-6.69, -2.90)***	-2.76 (-4.701, -0.82)***	-4.16 (-7.76, -0.56)**
**Medicine**	1.43 (0.25, 2.62)**	-0.97 (-1.99, 0.04)*	-0.48 (-0.98, 0.01)*	-0.04 (-0.50, 0.42)	-0.53 (-1.21, 0.14)	2.55 (-5.58, 10.68)	-0.90 (-3.03, 1.24)	-0.22 (-1.41, 0.96)	-0.40 (-1.49, 0.69)	-1.50 (-3.33, 0.36)
**Education**	-1.47 (-2.71, -0.24)**	0.45 (-0.61, 1.51)	0.47 (-0.13, 1.07)	0.02 (-0.58, 0.62)	-1.31 (-2.20, -0.43)***	-4.366 (-12.96) 4.232	1.61 (-0.70, .91)	1.24 (-0.16, 2.63)*	-0.24 (-1.53, 1.06)	-0.90 (-3.45, 1.66)
**Energy**	0.99 (0.11, 1.86)**	-0.69 (-1.43, 0.05)*	-0.89 (-1.29, -0.48)***	-0.93 (-1.31, -0.56)***	-0.09 (-0.57, 0.38)	-0.25 (-5.80, 5.30)	-2.34 (-3.93, -0.75)***	-2.74 (-3.77, -1.71)***	-1.79 (-2.62, -0.96)***	0.08 (-1.41, 1.56)
**Lifestyle**	0.17 (-0.40, 0.74)	0.41 (-0.06, 0.88)*	-0.04 (-0.29, 0.20)	-0.10 (-0.37, 0.18)	-0.19 (-0.62, 0.25)	0.75 (-3.63, 5.14)	1.13 (0.17, 2.09)**	0.00 (-0.58, 0.59)	-0.72 (-1.35, -0.08)**	-0.53 (-1.79, 0.73)
**Transportation**	-1.79 (-2.95, -0.63)***	-0.12 (-0.94, 0.69)	-0.47 (-0.98, 0.04)*	-0.98 (-1.50, -0.46)***	-1.03 (-1.88, -0.18)**	-3.40 (-11.13, 4.33)	1.19 (-0.60, 2.98)	0.53 (-0.74, 1.77)	-1.92 (-3.14, -0.69)***	-1.14 (-3.84, 1.56)
**Social Responsibility**	-0.59 (-1.43, 0.26)	0.14 (-0.67, 0.94)	0.00 (-0.44, 0.44)	0.10 (-0.42, 0.62)	-0.65 (-1.44, 0.13)	-1.47 (-8.63, 5.70)	-1.43 (-2.93, 0.07)*	-0.02 (-1.11, 1.07)	-0.31 (-1.34, 0.73)	-1.88 (-4.11, 0.35)*
**Durable Goods**	-0.16 (-0.63, 0.31)	-0.295 (-0.80, 0.21)	-0.12 (-0.38, 0.15)	-0.19 (-0.44, 0.06)	-0.42 (-0.85, 0.01)*	0.03 (-3.95, 4.01)	-0.56 (-1.56, 0.42)	0.19 (-0.64, 1.01)	0.51 (-0.12, 1.14)	-0.81 (-2.22, 0.61)

IQ^i^ indicates income quantile, where IQ-1 is the 1^st^ income quantile group, and IQ-5 is the 5^th^ income quantile group. * points out the probability value of the coefficients

^*^p < 0.10

^**^p < 0.05

^***^p < 0.01; 95% confidence intervals are in parenthesis

## Discussion

We estimated the crowding-out effect without controlling the covariates (unadjusted crowding out) and by controlling the covariates (adjusted crowding out). The unadjusted crowding-out effects reveal that expenditure on SSB crowds out the expenditure on food, clothing, housing, and social responsibility. In contrast, the adjusted crowding-out effects present that the expenditure on SSB crowds out the expenditure on housing, education, transportation, and social responsibility. In both cases, it is evident that SSB consumption displaces the household expenditure for basic necessities such as food, clothing, housing, transportation, and education. It implies that the consumption of SSB affects human capital development, and therefore, it hinders the well-being of the people. In a similar study on tobacco, Hussain et al. (2018) [18] also found that tobacco consumption reduces the expenditure on health and education, which affects human capital development and the well-being of the people.

The findings present that households with SSB consumption are more likely to have sick members who suffered from any chronic disease during the last twelve months. On the other hand, logistic regression results show that households with sick members are positively associated with consuming SSB or other sugar-added drinks. Unadjusted crowding out effects of SSB and sugar-added drinks depicts that the households that have expenditure on only soft drinks have greater expenditure on medicine, implying that SSB consumption poses a health burden. However, the coefficients for all specifications for adjusted crowding out are found positive, though they are not statistically significant. It insinuates that consumption of SSB increases the risk of being affected with chronic diseases, and therefore, it increases the expenses for medicine. It is evident that the consumption of SSB has also caused budget displacement in the household income portfolio.

Our econometric results also show that male-headed households are more likely to allocate their income to beverages and other sugar-added drink commodities. Therefore, crowding out for various food and non-food items is more prevalent for household consumption items in male-headed households. It seems rational because male members of the households remain mostly outside and therefore are more frequently used to beverages and sugar-added drinks than the female members.

The study has a number of limitations. As the latest HIES 2021 data is not publicly available yet, the study team analysed the HIES 2016 data. There might be changes in the SSB consumption pattern and related variables since 2016. Moreover, the available SSBs in Bangladesh are diverse in nature, and they are not well defined in Bangladesh for policy and practice. The HIES 2016 data also does not define 'SSB' explicitly. For instance, sherbats (like fruit juices) are added to the category of soft drinks (code 151) in the current HIES 2016. In addition, bottled waters are added with the soft drinks (code 194) in the dining out section. Therefore, it is not possible to disaggregate the sherbats from the soft drinks, or to determine whether the SSB or bottled water was consumed. In the absence of an operational definition of SSB and limitations of the available data, we have categorized the SSB in line with the questions asked in the HIES 2016 survey and their classification of SSBs. Despite the limitations, this study, for the first time, generates evidence of the crowding-out effect of sugar-sweetened beverages (SSBs) on household expenditure patterns in Bangladesh. We recommend that for future rounds of HIES, the SSB needs to be defined properly and collect the disaggregated response accordingly. This is also important for designing taxation on diverse types of SSB products and designing an intervention for all kinds of SSBs.

Since the consumption of SSB negatively affects the basic household consumption and well-being of the people, evidence-based policies are required in order to curb the use and overuse of SSB. Future research is needed to design and implement innovative interventions to curb the consumption of SSB.

## Conclusion

The increased consumption of sugar-sweetened beverages (SSBs) not only impacts human health but also crowds out the household income allocation to the basic necessities. Wielding nationally representative Household Income and Expenditure Survey (HIES) 2016 data, we found that male members, larger household sizes, household heads with higher education, profession, having a refrigerator, members living outside of the house, and households with higher income positively affect the decision of purchasing SSB. In addition, the consumption of SSB crowds out the household spending for basic necessities like food, clothing, housing, education, transportation, and social responsibilities. Based on our findings, we recommend evidence-based policies to regulate the sale and consumption of SSB for a healthy and productive nation.

## Data Availability

The datasets used and/or analyzed during the current study are available from the Household Income and Expenditure Survey, Bangladesh- [email: dg@bbs.gov.bd; web link: http://www.bbs.gov.bd/] on reasonable request.

## Footnotes

1 Please see the link for detail https://brooklyntea.com/blogs/news/tea-culture-in-bangladesh-1
